# Publisher Correction: Coherent diffractive imaging of single helium nanodroplets with a high harmonic generation source

**DOI:** 10.1038/s41467-017-02702-x

**Published:** 2018-01-16

**Authors:** Daniela Rupp, Nils Monserud, Bruno Langbehn, Mario Sauppe, Julian Zimmermann, Yevheniy Ovcharenko, Thomas Möller, Fabio Frassetto, Luca Poletto, Andrea Trabattoni, Francesca Calegari, Mauro Nisoli, Katharina Sander, Christian Peltz, Marc J. Vrakking, Thomas Fennel, Arnaud Rouzée

**Affiliations:** 1 Institut für Optik und Atomare Physik, Technische Universität Berlin, Hardenbergstraße 36, 10623 Berlin, Germany; 2 Max-Born-Institut für Nichtlineare Optik und Kurzzeitspektroskopie, Max-Born-Straße 2A, 12489 Berlin, Germany; 3 European XFEL GmbH, Holzkoppel 4, 22869 Schenefeld, Hamburg Germany; 4 CNR, Istituto di Fotonica e Nanotecnologie Padova, Via Trasea 7, 35131 Padova, Italy; 5 Center for Free-Electron Laser Science, DESY, Notkestr. 85, 22607 Hamburg, Germany; 6 CNR, Istituto di Fotonica e Nanotecnologie Milano, Piazza L. da Vinci 32, 20133 Milano, Italy; 7 Department of Physics, Politecnico di Milano, Piazza L. da Vinci 32, 20133 Milano, Italy; 8 Institut für Physik, Universität Rostock, Albert-Einstein-Straße 23, 18059 Rostock, Germany

Correction to: *Nature Communications* 10.1038/s41467-017-00287-z, Article published online 08 September 2017

In the original version of this Article, the affiliation for Luca Poletto was incorrectly given as ‘European XFEL GmbH, Holzkoppel 4, 22869 Schenefeld, Hamburg, Germany’, instead of the correct ‘CNR, Istituto di Fotonica e Nanotecnologie Padova, Via Trasea 7, 35131 Padova, Italy’. This has now been corrected in both the PDF and HTML versions of the Article.

