# Supplementary material for: Coherent diffractive imaging of single helium nanodroplets with a high harmonic generation source
Source: Nat Commun. 2017 Sep 8;8:493. doi: 10.1038/s41467-017-00287-z (PMC5591197; doi:10.1038/s41467-017-00287-z)
Supplement: Supplementary file 1 — Supplementary Information [file 41467_2017_287_MOESM1_ESM.pdf]

File name: Supplementary Information
Description: Supplementary figures and supplementary notes.

File name: Peer review file
Description:

Supplementary Note 1) Multicolor Mie fits using the literature values of bulk Helium

The input parameters for the multidimensional Mie-based optimization are the particle size, the refractive indices at the wavelengths of the contributing harmonics, the relative intensities of the harmonics, and the intensity of the XUV pulse. We find that using the literature values for the refractive indices of bulk liquid helium at the harmonic wavelengths as fixed input parameters does not allow fitting the measured diffraction patterns. This claim is supported by the results of two attempts to fit the data. First, only the particle size and the total XUV intensity were varied, while the relative harmonic intensities were fixed to the average measured spectrum (cf. Fig 3d, main manuscript) and the literature values of the refractive indices at the harmonic wavelengths (n_{11}=0.97 + 0.0i; n_{13}=1.14+0.032i; n_{15}=1.03+0.029i; n_{17}=0.9964 + 0.041i; see Methods) were used. As a second test, we also allowed the optimization of the intensities of the individual harmonics, as discussed below.

Typical results for the first scenario are shown in Supplementary Figure 1 for three measured profiles (black) derived from a representative ring-type scattering pattern. The optimization with fixed refractive indicies and fixed relative harmonic intensities (green) is unable to provide a reasonable fit of the measured data. Particularly large discrepancies to the measured profile remain at high scattering angles. For comparison, all qualitative features of the profiles can be fitted when the refractive indicies of the 13th and 15th harmonic are included in the optimization (purple curve).

For the second test, the refractive indices were kept fixed to the literature values but the intensity ratios of the harmonics were freely varied in addition to the optimization parameters droplet size and total XUV intensity. The resulting fits are displayed in Supplementary Figure 2. A better match between the measured profiles (black) and the calculated profiles (purple) can be obtained compared to the first test shown in Supplementary Figure 1. However, the residual deviations remain substantially larger when compared to the results obtained with optimized optical parameters and fixed intensity ratios, compare black and red/blue symbols in Supplementary Figure 3b. Moreover, the intensity ratios between the harmonics retrieved from the fitting procedure (see Supplementary Figure 3a) are in stark contrast to the measured spectrum (cf. Fig. 3d of the main manuscript), where the 13th and 15th harmonic have the strongest contributions (blue and red). Here, the most intense contribution given from the fits originates from the 17th harmonic that has been measured to be the weakest. We can therefore conclude that the refractive indices for Helium nanodroplets are different from the reported values of bulk liquid helium.

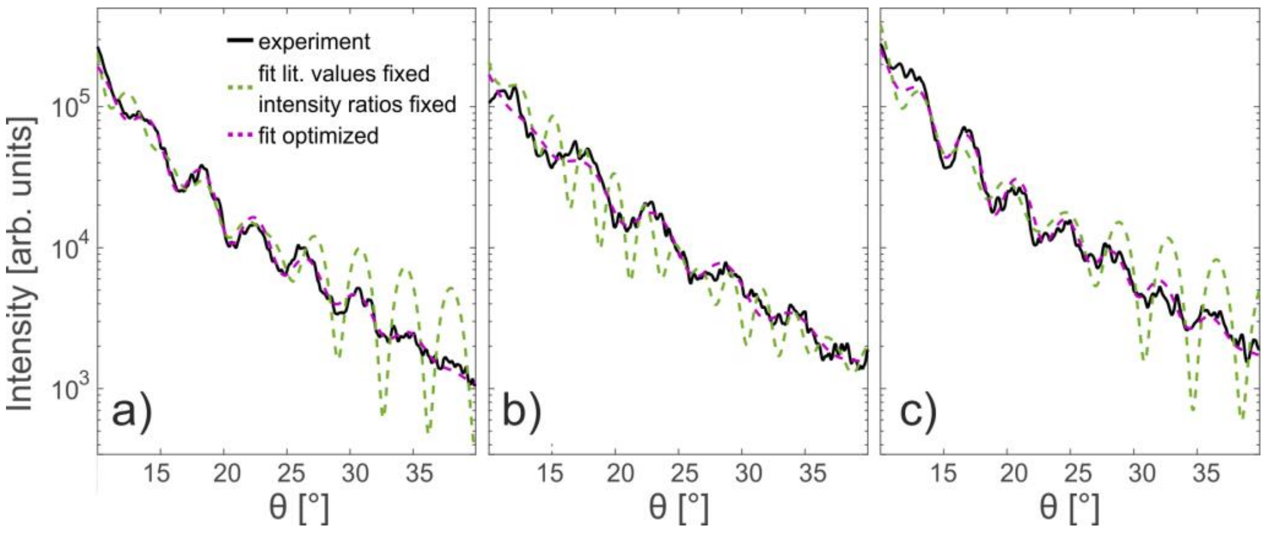

Supplementary Figure 1: Multicolor Mie fits with fixed intensity ratios and refractive indices. Black curves are radial intensity profiles retrieved from three recorded scattering patterns (a-c) , green dashed curves display best fit obtained with the available refractive index data for liquid bulk helium and fixed relative harmonic intensities. The results are compared to the optimization with variable refractive indicies for the 13th and 15th harmonic (purple dashed lines) as used in the main text.

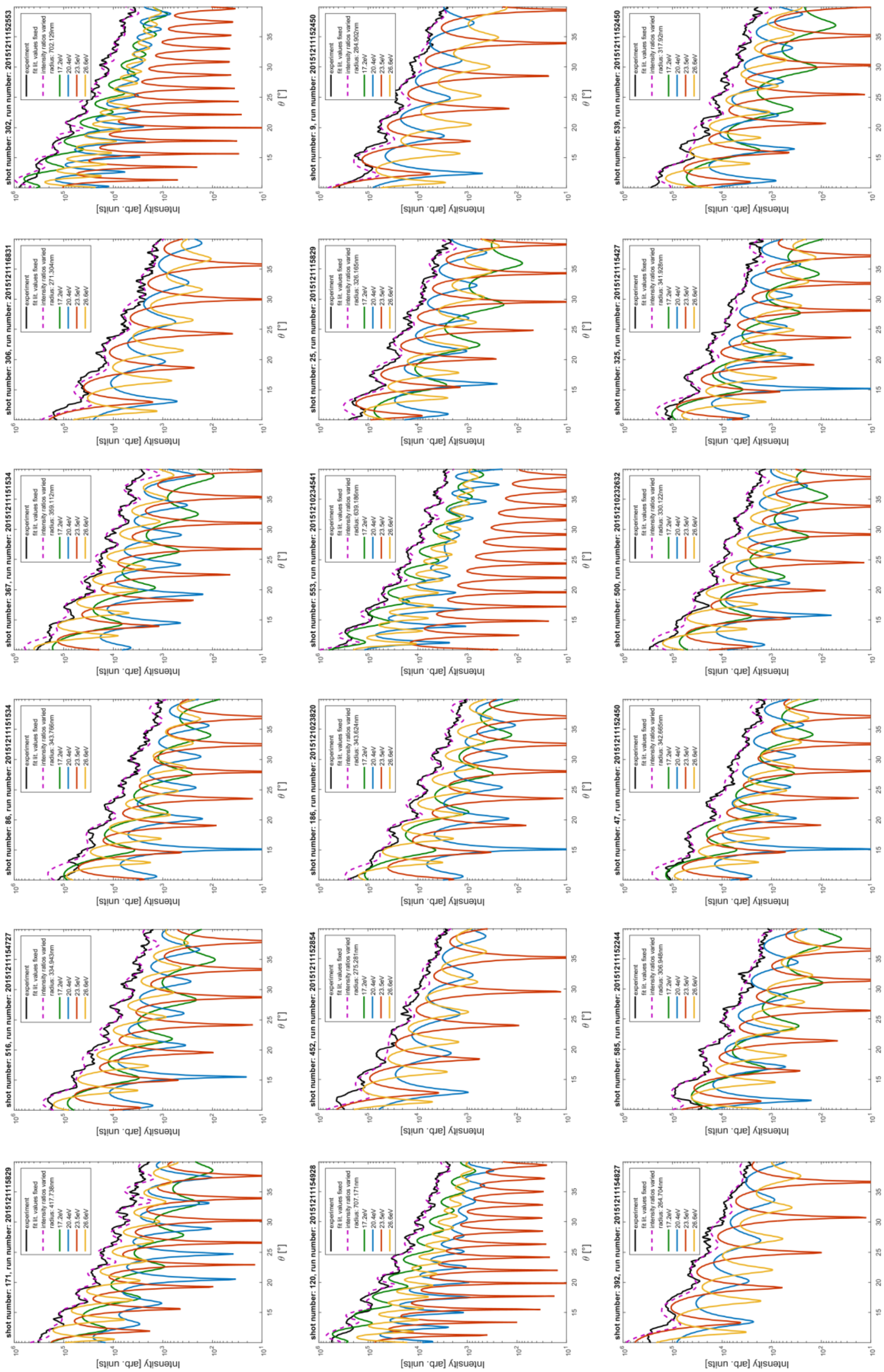

Supplementary Figure 2: Fits with open intensity ratios. Radial intensity profiles retrieved from 18 experimentally obtained bright diffraction patterns (black). The corresponding multicolor Mie fits (purple curves) are obtained considering the refractive indices fixed to the literature values while freely optimizing the intensity ratios between harmonics. The contributions from individual harmonics obtained in the fit are color coded as indicated in the legend.

Supplementary Figure 3: Assessment of fits with open intensity ratios. a) Weight of 11th (green), 13th (blue), 15th (red) and 17th (yellow) harmonics obtained from the multicolor Mie-based fitting routine when using the refractive indices of the bulk values of liquid helium and varying the intensity ratios between harmonics as fitting parameters. b) Residual errors (defined as mean square displacement of the logarithmic intensity profiles) for all optimized patterns in Supplementary Figs. 2, 5 and 6. Black: Refractive indices fixed to literature values, intensities free. Blue: Solution with 13th harmonic dominant. Red: Solution with 15th harmonic dominant.

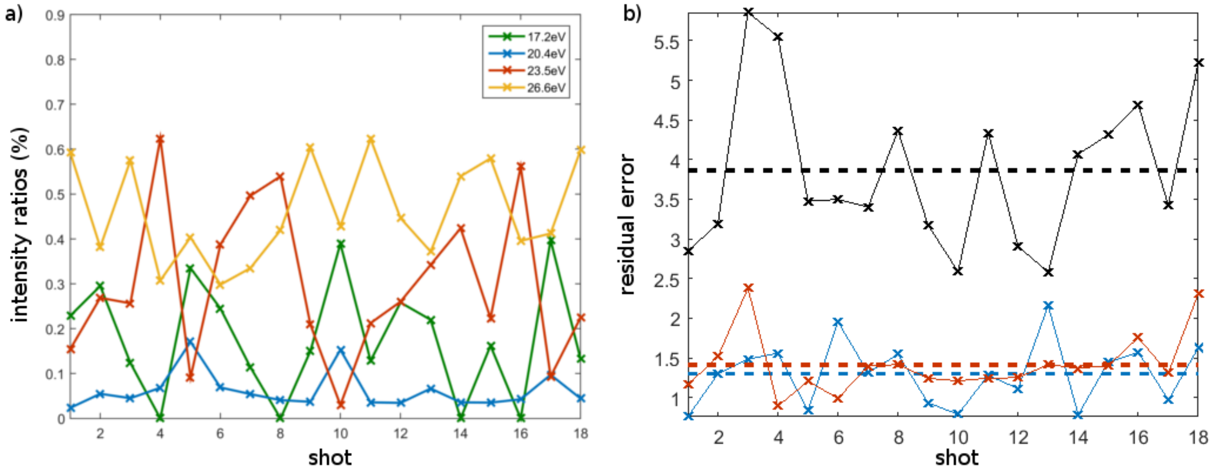

Supplementary Note 2) Solutions with comparable residuals

As described in the main manuscript, best fits were achieved when varying the refractive indices of the most intense harmonics 13 and 15, while the relative harmonic intensities were set to the average measured values and the refractive indices at the wavelengths of the 11th and 17th harmonics were fixed to the literature values.

Under these conditions, the majority of the best fits for the 18 recorded scattering profiles leads to refractive indices that lie around an average pair of values: n_{13} =0.925+ 0.018i; n_{15} = 1.269+0.042i, see Supplementary Figure 4a. In these fits, the 13th harmonic gives the dominant contribution to the diffraction image (Supplementary Figure 5). However, for all analyzed patterns, a second solution is found by the algorithm where the dominant contribution systematically comes from the 15th harmonic (Supplementary Figure 6). The refractive indices obtained by the second solution are shown in Supplementary Figure 4b, here the results cluster around another value pair (n_{13} =1.2827+0.0464i; n_{15} = 0.9518+0.0269i). The straight lines indicate the corresponding value pairs of single fit results. The fitting errors for these two systematic solutions are compared in Supplementary Figure 3b to the results obtained for open intensity ratios and refractive indices fixed to the literature bulk values. While it is clear that varying the refractive indices leads to a substantial reduction of the residual errors, the quality of the two systematic solutions is similarly good. We note that the refractive indices for the solution with dominant 15th harmonic lie closer to the literature values. However, considering the signal to noise ratio in our experiment, we cannot fully exclude one of the two solutions. Additional experiments using a XUV pulses with larger distances between the harmonics and/or with a better signal to noise ratio are required to resolve this ambiguity.

Supplementary Figure 7 displays histograms of the resulting droplet sizes from the optimization. Comparing Supplementary Figure 7a and b shows that the algorithm, while interchanging the roles of 13th and 15th harmonic between the two solutions, also adjusts the droplet size accordingly to account for the different wavelength of the dominant harmonic (the distributions are shifted by approximately 50 nm). We note that the size distribution of the droplet jet is probably broader than indicated by the histogram, because droplets with sizes outside the observed range (250 nm - 550 nm) cannot be fitted. Smaller droplets produce a too dark diffraction pattern and very large droplets have a very small fringe separation such that minima are more likely contaminated by noise fluctuations.

However, despite the remaining ambiguity, the results support that the multicolor approach in principle allows the systematic characterization of the properties of individual droplets within a wide size distribution.

Supplementary Figure 4: Refractive indices from fits. Blue circles correspond to the 13th harmonic and red circles to the 15th harmonic. (a) Results from multicolor Mie fits in Supplementary Fig. 5, when the 13th harmonic gives the dominant contribution and (b) from Supplementary Fig. 6, when the 15th harmonic is dominant. The solutions for the fits presented in Fig. 2 of the main manuscript are indicated by squares, literature refractive indices for bulk liquid helium are indicated by stars.

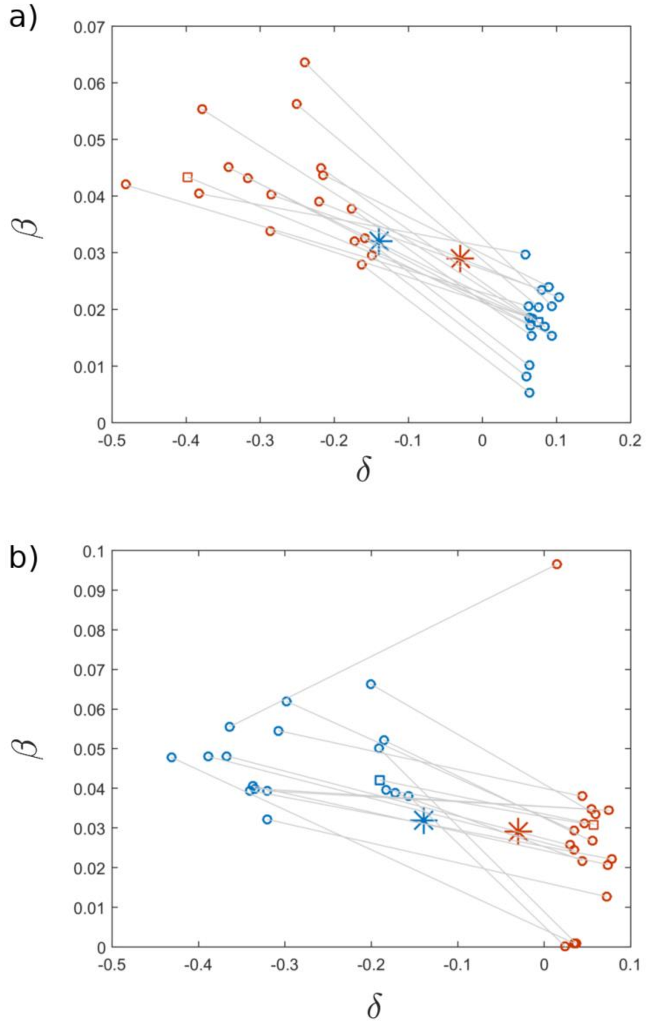

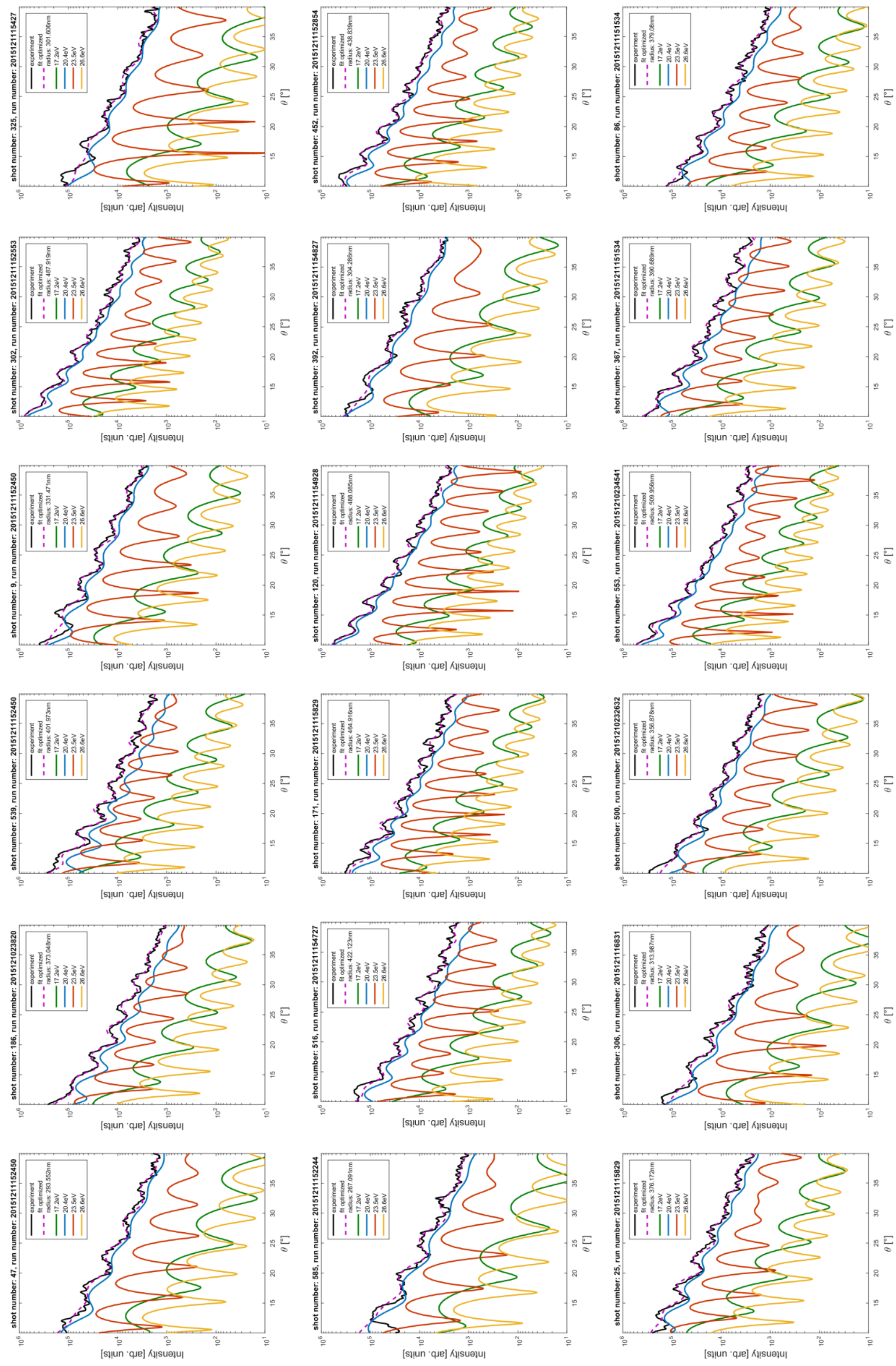

Supplementary Figure 5: Fits with dominant 13th harmonic contribution. Radial intensity profiles of 18 experimentally measured, bright scattering patterns in black, fits in purple, harmonic contributions color coded.

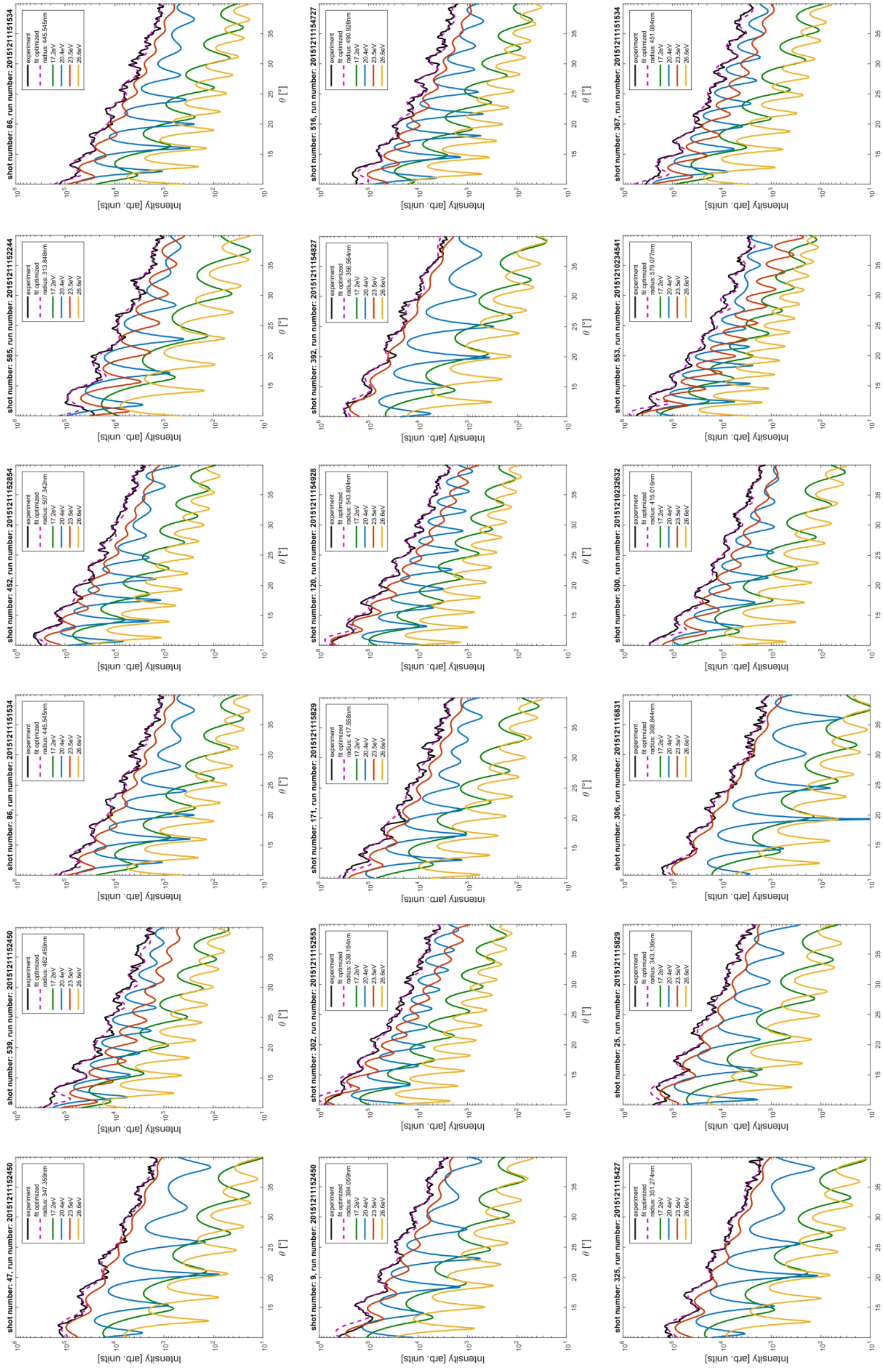

Supplementary Figure 6: Fits with dominant 15th harmonic contribution. Radial intensity profiles of 18 experimentally measured, bright scattering patterns in black, fits in purple, harmonic contributions color coded.

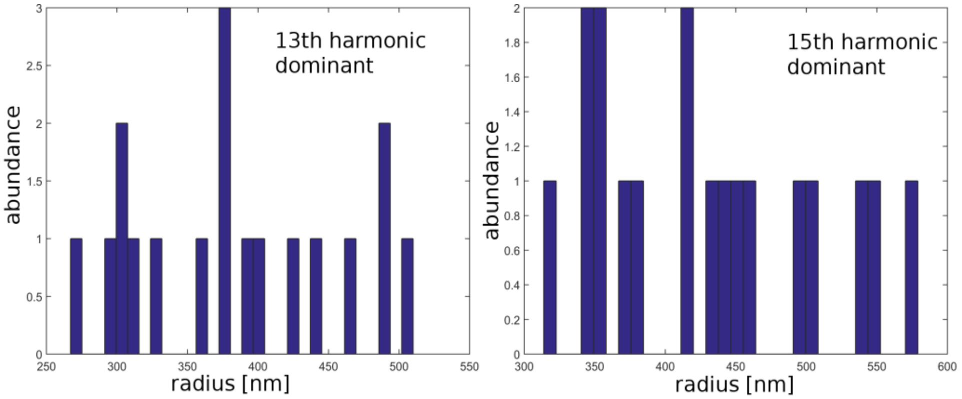

Supplementary Figure 7: Size distribution for all best fits. a) Fits where the 13th harmonic gives the dominant contribution. Average radius <*R*>=383 nm. b) Size distribution for 15th harmonic dominant. Average radius <*R*>=430 nm.

Supplementary Note 3) Statistical considerations of straight and bent streak patterns

In our experiment, most of the observed streaked patterns exhibit a bending with varying degree of curvature (in total 68 patterns with streaks were observed, among them 7 straight ones). Simulations considering prolate, pill-shaped droplets (see Supplementary Figure 8, only a single harmonic was considered for simplification and the calculation was carried out in 1st Born approximation) show that tilt angles between 10° and 80° should create clearly observable bent streaks in the diffraction patterns (here the tilt angle is defined between the semi-minor axis of the prolate particle and the optical axis). Below 10° tilt angle, the streaks would appear to be straight. Tilt angles above 80° do not create clear streak features and are therefore not contained in the statistics given above. As a result of these considerations, one would expect that up to 1/8 (0-10° out of 0-80°) of the patterns should display straight streaks and 7/8 exhibit bent streaks when assuming rod-like structures with random orientation (only the angle between the symmetry axis of the structure and its projection on the scattering plane is important to draw this conclusion, as (i) the shapes have a cylindrical symmetry and (ii) a rotation around the axis of the incident beam only leads to a corresponding rotation of the pattern). The statistics suggested by Supplementary Figure 8 are in reasonable agreement with our observations.

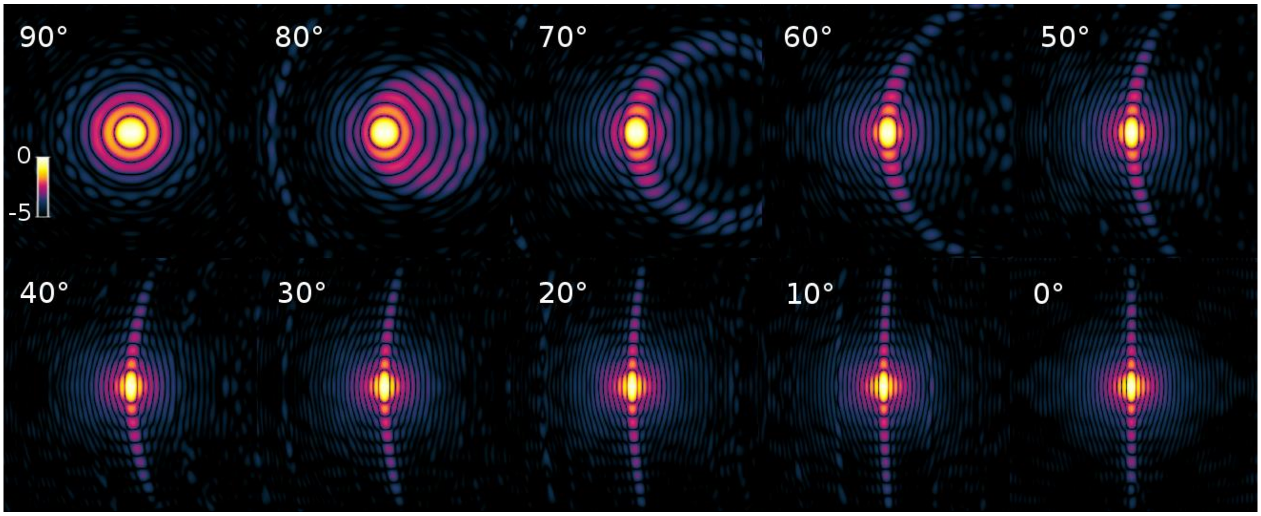

Supplementary Figure 8: Simulation of a prolate pill-shaped structure at different orientations. Parameters of the single color wide angle diffraction patterns: Long axis/short axis = 3, tilt angle defined between semi-minor axis of prolate particle and XUV beam (tilt angle indicated in each pattern). Arbitrary intensities, logarithmic color scale.
